# Meeting a need: development and validation of PubMed search filters for immigrant populations

**DOI:** 10.5195/jmla.2024.1716

**Published:** 2024-01-16

**Authors:** Q. Eileen Wafford, Corinne H. Miller, Annie B. Wescott, Ramune K. Kubilius

**Affiliations:** 1 q-wafford@northwestern.edu, Research Librarian, Galter Health Sciences Library & Learning Center, Northwestern University Feinberg School of Medicine, Chicago, IL; 2 corinne.miller@northwestern.edu, Clinical Informationist, Galter Health Sciences Library & Learning Center, Northwestern University Feinberg School of Medicine, Chicago, IL; 3 annie.wescott@northwestern.edu, Research Librarian, Galter Health Sciences Library & Learning Center, Northwestern University Feinberg School of Medicine, Chicago, IL; 4 r-kubilius@northwestern.edu, Collection Development/Special Projects Librarian, Galter Health Sciences Library & Learning Center, Northwestern University Feinberg School of Medicine, Chicago, IL

**Keywords:** Filter, filters, immigrant health, search strategy, filter development, hedge, hedges

## Abstract

**Objective:**

There is a need for additional comprehensive and validated filters to find relevant references more efficiently in the growing body of research on immigrant populations. Our goal was to create reliable search filters that direct librarians and researchers to pertinent studies indexed in PubMed about health topics specific to immigrant populations.

**Methods:**

We applied a systematic and multi-step process that combined information from expert input, authoritative sources, automation, and manual review of sources. We established a focused scope and eligibility criteria, which we used to create the development and validation sets. We formed a term ranking system that resulted in the creation of two filters: an immigrant-specific and an immigrant-sensitive search filter.

**Results:**

When tested against the validation set, the specific filter sensitivity was 88.09%, specificity 97.26%, precision 97.88%, and the NNR 1.02. The sensitive filter sensitivity was 97.76%when tested against the development set. The sensitive filter had a sensitivity of 97.14%, specificity of 82.05%, precision of 88.59%, accuracy of 90.94%, and NNR [See Table 1] of 1.13 when tested against the validation set.

**Conclusion:**

We accomplished our goal of developing PubMed search filters to help researchers retrieve studies about immigrants. The specific and sensitive PubMed search filters give information professionals and researchers options to maximize the specificity and precision or increase the sensitivity of their search for relevant studies in PubMed. Both search filters generated strong performance measurements and can be used as-is, to capture a subset of immigrant-related literature, or adapted and revised to fit the unique research needs of specific project teams (e.g. remove US-centric language, add location-specific terminology, or expand the search strategy to include terms for the topic/s being investigated in the immigrant population identified by the filter). There is also a potential for teams to employ the search filter development process described here for their own topics and use.

## INTRODUCTION

Immigrants are individuals who, for various reasons, left their country of birth and reside or resided in a different country. The worldwide increase in immigration and individuals identified as immigrants corresponds to a rise in studies on immigrant health. As of November 2022, over 450 systematic reviews had been published in PubMed in the preceding five years on immigrant health. The topics included social determinants of health in immigrant populations, public health interventions for immigrants, health trajectories of immigrants, and the impact of migration on health status disparities [1–4]. As librarians and information professionals, we have experienced many challenges in searching for studies about immigrants. Existing filters including the “Immigrant Health Disparities” filter, which our project team developed in 2018 and 2019, vary in comprehensiveness and many have limited information on the methods used to generate the terms and the overall performance of these filters [5–10]. We launched the development and validation of PubMed search filters to meet our identified information needs, and those of our patrons, for retrieval of peer-reviewed literature on immigrant populations. Subsequently, our team embarked on a multi-year, multifaceted filter development process which allowed us to address a wide range of concepts and challenges.

To start, it was challenging but essential to define the population covered by our project scope due to the complexity of immigrant populations and how immigrants are studied and described in scholarly research. For example, immigrants and immigration may be key subjects in studies on language, culture, race, and ethnicity; however, these studies may never use immigrant-explicit terminology. Loetscher et al explore the influence of immigration on pregnancy outcomes in Switzerland [11]. The study conveys immigrant status using the phrase “mothers from” in conjunction with a foreign country. The record in PubMed uses no other explicit terms such as “immigrants” and “migrants” to describe the population. We also recognized that immigrant and immigration are evolving and sensitive topics, with nuances that influence term selection, the introduction or disuse of terms (e.g., “illegal immigrants”), and the overrepresentation of US-based research.

Perceptions of immigrants present their own set of challenges. Some groups identify “immigrants” as being residents from a different country [5]. However, the perception of foreignness is more nuanced due to internal migration as well as geopolitical and historical events. For example, “diaspora” can be associated with established populations such as Black Americans in the United States or more recent diasporic events such as Syrians immigrating to European countries. In response to these challenges, we dedicated substantial time and effort to defining immigrants during the project's scope and eligibility criteria phase.

Our examination of various term-generating strategies employed by teams developing comprehensive search filters revealed diverse strategies. Many teams generated terms using word frequency analysis and text analysis software such as PubReminer, Wordstat, Simstat, Concordance, and VOSViewer [12–20]. This approach often requires a manual review of the terms. A growing number of teams relied solely on automated processes, including data visualization tools and frequency analysis, to analyze user data with statistical modeling [17, 21]. Other teams relied on clinician, librarian, and expert opinions or recommendations to generate relevant terms [13, 20, 22]. Several teams undertook manual assessment or other unspecified approaches in reviewing relevant records and identifying relevant titles, abstracts, or controlled vocabulary terms [22–28]. Some teams combined manual review with automated processes, such as frequency analysis or applying an existing filter as a starting point to identify relevant articles [29, 30].

While we examined and used existing filter development processes as a foundation, the complexity of the topic led us to adapt our methodology. We initially aimed for a single filter for research on immigrants in PubMed, however, we realized the two-filter approach would give researchers the option to pull immigrant-related studies that use language and culture words to describe immigrants. Overall, the resulting sensitive and specific filters complement existing filters while providing reproducible methods and performance outcomes that are comparable with other comprehensive filters.

## METHODS

**Figure 1:**
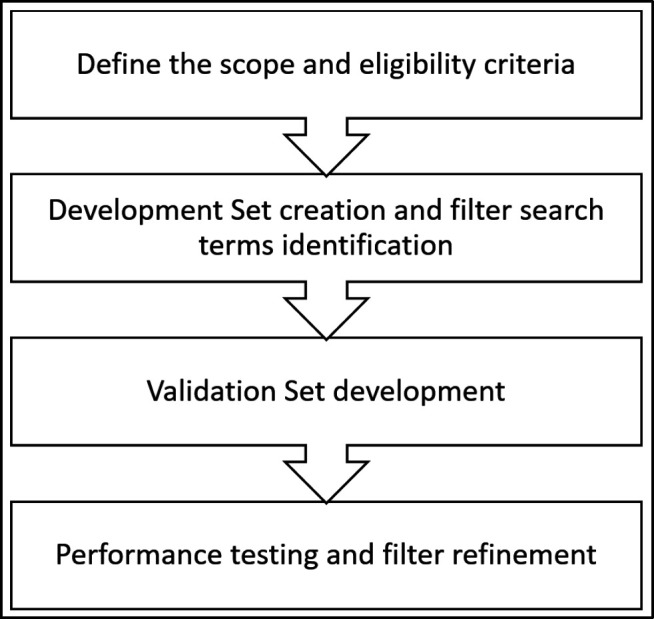
Filter Development Phases

Our methodologic approach relied primarily on the following four key phases (also illustrated in Figure 1):

1. Established a clear scope and eligibility criteria. This meant defining the population, establishing the inclusion and exclusion criteria for both studies and search terms, as well as compiling a list of known immigrant search terms from authoritative sources.
2. Created a development set or “gold standard” set comprised of references that meet the inclusion criteria, which the team reviewed, then extracted the immigrant-related term or terms to create the immigrant search filters for PubMed.
3. Created a validation set of references. These were separate sets of references that meet the inclusion criteria and were used to test the filters.
4. Conducted performance tests and revised the search filters as needed. We tested the filters against the validation set references and revised filters to optimize performance.

We compiled search terms for both filters by combining terms derived from authoritative sources (subject experts as well as academic and government publications), the development (gold standard) set references, and references identified from the validation set reference after we conducted the performance tests. As other filter development teams did, we applied manual review and automation to generate the filter terms.

We reviewed different approaches to validating or testing the filters. External validation is described by the UK InterTasc Information Specialists' Sub-Group (ISSG) as testing the filters against a set of records distinct from those used in the filter development [31]. We found that most filter development teams used external validation [13, 15–18, 21, 24, 26, 29, 30, 32–39]. Fewer teams opted for internal validation and tested the filter against the development set of records used to generate the filter [19, 27, 28, 40, 41]. We employed external validation by testing the specific and sensitive filters against the validation set. We selected external validation because we believed testing the final filter or filters against a separate set of references added a layer of objectivity because they were tested against a unique, yet relevant set of references.

We expand on our methodology in the subsequent text and highlight key methodological terms in Table 1.

**Table 1:** Glossary of project methodological terms

Term	Definition
Development set references	References that meet the inclusion criteria and are assessed to create the filter search terms. Also called the gold standard set.
Eligibility criteria	A predefined set of criteria applied to articles and terms during the screening process to determine whether they will be included or excluded for use in the search filter.
Exclusion criteria	A predefined set of criteria that disqualifies prospective articles or terms from the filter.
Inclusion criteria	A predefined set of criteria that must be present to be included in the search filter.
Indicator	Qualifier or companion word or words, usually non-immigrant specific, that must be combined with another term to indicate immigrant population and retrieve the relevant reference.
Sensitive filter	Search filter with the specific, language and culture terms.
Snowball set references	Studies from the reference lists of relevant systematic reviews identified from the initial topic search in PubMed. These references were randomly divided into two sets, one for the development set and the other for the validation set.
Specific filter	Search filter with specific terms that do not require an indicator.
Validation set references	References known to meet the inclusion criteria and used to test the performance of the search filter.

### Defining the Scope and Eligibility Criteria

During the development of the “Immigrant Health Disparities” search filter in 2019, we queried experts with research focused on immigrant health for recommendations on terms and definitions for immigrants. The experts' input guided us toward the definition by Diaz et. al, which describes immigrants as “persons who are moving or have moved across an international border away from their habitual place of residence, regardless of the causes for the movement or the voluntariness of their decision” [8]. This definition informed our discovery process and served as an essential point of reference in defining our scope. The eligibility criteria were applied during all phases of the project.

As part of our inclusion criteria, we included any study that used immigrant-explicit terminology such as “immigrant” or “refugee.” This includes individuals of foreign origin regardless of their immigration status (e.g., nonimmigrant workers). We included relevant studies regardless of geography. For example, studies about immigrants living in Sweden or China met the inclusion criteria. Language presented a unique challenge because many non-immigrants may speak a second language or multiple languages. Although individuals who do not identify as immigrants may speak a language different from the general population, many researchers use language phrases and concepts to communicate immigrant status. Consequently, we elected to include records with linguistic-related terms that conveyed a foreign or perceived non-native population such as “non-English speaking” or “language barriers.” We encountered a similar challenge with culture as many medical research studies equate cultural differences and acculturation with immigration. We marked references that used culturally specific terms and specific populations for inclusion in our filter and analysis.

**Exclusion Criteria:** Our exclusion criteria included studies that presented individuals and national, demographic, or administrative geographical units, without conveying international movement. We excluded studies that solely examined individuals' health based on race and ethnicity without taking into account their immigration status, as there are already comprehensive filters for race and ethnicity [42]. We did not restrict studies based on language or place of publication, but in the case of non-English references, relied on the translated title and English language abstract in PubMed.

### Development Set (Gold Standard) References

The development set encompasses known references that meet the inclusion criteria [25]. To form the development set reference list, we ran a topic search in PubMed MEDLINE for systematic reviews on preventive health or pregnancy, both search topics were commonly requested by our patrons. This search resulted in 14,095 records, which we divided into two screening sets for two reviewer teams who then independently screened the titles and abstracts of their assigned set in Rayyan, the screening platform we used for the project. We resolved conflicts through discussion between all four reviewers, which resulted in 135 references meeting the inclusion criteria. From the reference lists of these systematic reviews, we then identified 4,531 unique records indexed in PubMed MEDLINE. This created the snowball set of references as highlighted in Table 1. The snowball set of references helped us populate the development and validation sets with potentially relevant references. We randomly selected half (2,266) of the references in the snowball set and assigned them to the development set for screening using the inclusion criteria. We set aside the other half of the snowball set references (2,265 records) to build the validation set references.

After screening titles and abstracts, we identified 894 of the 2,266 studies to include in the development set. We transferred the titles and available abstracts for each record in the development set to a spreadsheet. We separated those records into four groups, one for each team member to extract terms and phrases that met the inclusion criteria. The relevant terms or phrases were extracted and ranked based on the three-tiered system outlined in Table 2. Team members independently reviewed each term according to the inclusion criteria. We addressed conflicting rankings through discussion and consensus. We ensured that all included terms and phrases were in the PubMed Index [43].

**Table 2:** Ranking system

Rank	Description
1	Terms met inclusion criteria without the need for an indicator or indicators.
2	Terms met the inclusion criteria when paired with a single indicator.
3	Terms required multiple indicators to meet the inclusion criteria.

Rank 1 terms met the inclusion criteria without the need for an additional indicator or indicators to describe immigrants or immigration. We combined Rank 1 terms with the words derived from the expert consensus and authoritative sources to produce the specific filter. As is the nature of certain terms, there are instances where Rank 1 terms, specific terms (e.g., migration, migrated), are commonly used in the literature to describe biomedical processes. Terms were considered and tested before inclusion. When the terms were tested alone against the validation set, the exclusion of “migrated” resulted in a 1% loss rate while the exclusion of “migration” resulted in an 12% loss rate. Further, when we tested the terms exclusion from the search filter when applied our validation set, the exclusion of “migrated” resulted in no change to the filter's recall, while the exclusion of “migration” resulted in a loss of recall. The term “migration,” therefore, was included in the final filter, while “migrated” was not included.

If a term needed a single indicator, we moved it to Rank 2. We placed terms requiring multiple indicators to communicate immigrant or immigration under Rank 3. The variability in Rank 3 words' indicators made it unfeasible to incorporate those words. As Rank 2 terms required clarification through the inclusion of an indicator, we tested each Rank 2 term or phrase against the specific search string. We assessed the number of unique results identified by that term and not by the specific search filter. We reviewed those unique records for relevant publications and determined inclusion if the term generated relevant records.

Indicators for Rank 2 terms fell under race and ethnicity, culture, language, and geographical location. Several records with Rank 2 terms had indicators from multiple categories. Given the complexity of immigrants and immigration and the use of language and culture to describe immigrants, we felt it necessary to incorporate both concepts by creating a sensitive search filter. The sensitive filter combines all the immigrant terms as well as terminology for language and culture terms. This produced a second filter with greater sensitivity or ability to retrieve all relevant studies because it is a broader search. The language and culture terms came from our term-extraction process and existing search strings [6, 7, 44–64].

To generate the Medical Subject Headings (MeSH) terms, we input the existing immigrant terms and PMIDs of the development set into the PubReminer software [65]. We also input terms into the MeSH browser for additional words [66]. We manually reviewed the results from the PubReminer and MeSH Browser queries for relevancy and inclusion. In 2022, we reran the search in PubReminer and scanned the MeSH browser for changes and new related headings. The specific and sensitive PubMed search filters are available in Appendix A.

### Validation Set References

The validation set references are relevant studies the immigrant population filters should find in PubMed. We created the validation set by combining the 2,266 references from the snowball set with the 1,270 unique PubMed records from the Journal of Immigrant and Minority Health, a prominent peer-reviewed journal in the field, as well as its predecessor, the Journal of Immigrant Health. We performed the journal search on May 20, 2020, and an updated search on June 9, 2022, which produced 1,266 records. In all, we screened 4,802 unique records to determine inclusion in the validation set. We divided the records into two sets for an independent title and abstract screening by two pairs of team members. A total of 2,830 records met criteria for inclusion in the validation set after resolving conflicting decisions through discussion and consensus. Once completed, the validation set acted as a sample for testing both the full search filter and individual terms to determine various performance measures of the filter as a whole (e.g. how many of our validation set records were captured by our two filters) and individual terms (e.g. testing the recall of a specific term to determine inclusion).

**Table 3:** Definitions of and formulas for performance measures

Performance measure	Definition/Formula
Correct Inclusion	Relevant records retrieved by the filter, true positives.
Incorrect Inclusion	Irrelevant records retrieved, false positives.
Correct Exclusion	Irrelevant records not retrieved, true negatives.
Incorrect Exclusion	Relevant records not retrieved, false negatives.
Sensitivity	The number of relevant records retrieved determined by (correct inclusion) / the total number of relevant records (relevant records).
Specificity	The proportion of irrelevant records not retrieved calculated by (correct exclusion) / (irrelevant records).
Precision	The proportion of retrieved records that are relevant calculated by (correct inclusion) / (total records retrieved).
Accuracy	The proportion of all records correctly included or correctly excluded determined by (correct inclusion + correct exclusion) / (all records screened).
Number Needed to Read	The number of records that need to be read in order to identify a single relevant result calculated by 1 / (precision).

## RESULTS

We tested the performance of the specific and sensitive filters in PubMed on September 22, 2022.

The sensitive filter generated 1,674,705 results and captured 874 of the 894 references in the development set. This filter missed 20 relevant studies. Two missed studies used relevant phrases (e.g., “foreign in-home workers” and “born in a country with”) that were not in the PubMed Phrase Index. One study required the use of “generation” and variants of the terms. Nine records required a specific country or geographical name, five required a specific language, and two references had specific languages with specific ethnicities. The sensitivity of the sensitive filter was 97.76% when tested against the development set.

**Figure 2:**
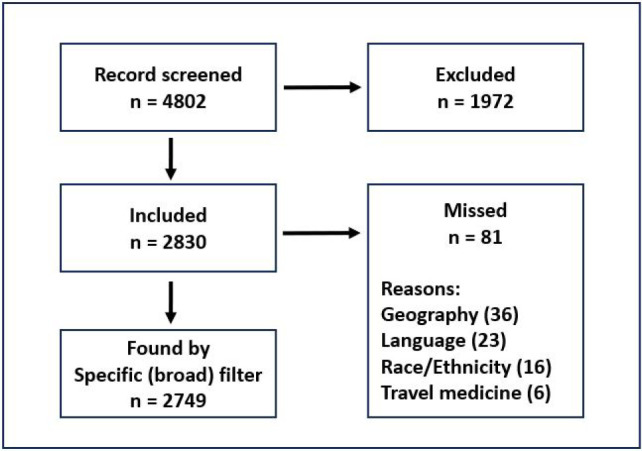
Flowchart for the creation of the validation set

The sensitive filter correctly captured 2,749 references (correct inclusion) from the validation set and missed 81 references (incorrect exclusion) that met the inclusion criteria. This contributed to a sensitivity of 97.14%. Records were missed because they used terms for geographic locations (e.g., “born in Mexico”), specific language terms (e.g., “Spanish”), specific race and ethnicity terms (e.g., “Korean American”), or terms related to travel medicine. The number of records missed for each reason is shown in Figure 2.

The sensitive filter pulled 354 references that did not meet the inclusion criteria (incorrect inclusion), and it correctly excluded 1,618 irrelevant references (correct exclusion) contributing to a specificity of 82.05%. The sensitive filter performed with a precision of 88.59%, accuracy of 90.94%, and NNR of 1.13.

The specific filter yielded a total of 460,584 results. When tested against the 894 references in the development set, the specific filter correctly identified (correct inclusion) 779 of the 894 references. It excluded 115 relevant references (incorrect exclusion). Overall, the sensitivity of the specific filter when tested against the development set was 87.14%.

When tested against the validation set, the specific filter correctly found 2,493 references of the 2,830 references in the validation set (correct inclusion). It failed to include 337 references that met the inclusion criteria (incorrect exclusion). This resulted in a sensitivity of 88.09%. The filter incorrectly included 54 references that did not meet the inclusion criteria (incorrect inclusion). It correctly excluded 1,918 references that did not meet the inclusion criteria (correct exclusion), which resulted in a specificity of 97.26%. In all, the specific filter produced a precision of 97.88%and an accuracy of 91.86%. Accuracy was determined by the records correctly included or correctly excluded. The number needed to read (NNR) was 1.02.

**Table 4:** Performance of the search filters

Filter	Correct Inclusion	Incorrect Inclusion	Correct Exclusion	Incorrect Exclusion	Sensitivity (%)	Specificity (%)	Precision (%)	Accuracy (%)	NNR
Sensitive (broad) vs Validation Set	2749	354	1618	81	97.14	82.05	88.59	90.94	1.13
Specific (focused) vs Validation Set	2493	54	1918	337	88.09	97.26	97.88	91.86	1.02

While we did not have a baseline or separate comprehensive immigrant population filter to compare performance measurements against, the sensitivity and specificity of the Immigrant Population filters closely resemble numbers generated by the Clinical Study Categories search filters to identify therapy studies and randomized controlled trials in PubMed [22]. PubMed's Therapy filter optimized for sensitive/broad search has a sensitivity of 99% compared to our sensitive filter sensitivity of 97%. The specificity for the sensitive filter was 70% for PubMed and 82% for the sensitive filter. The Haynes team's' Therapy filter optimized for specific/focused or narrower searching resulted in a sensitivity of 93% and specificity of 97%. The specific filter produced a sensitivity of 88% and specificity of 97%. We believe we found a balance between sensitivity and specificity because higher sensitivity indicates less likelihood of missing relevant literature. Higher specificity means less likelihood of retrieving irrelevant records and is inversely related to sensitivity.

## DISCUSSION

A comprehensive filter to find studies related to immigrant populations is essential for both healthcare providers working with immigrant and refugee populations, and for researchers seeking to learn more about the topic [67]. Initially, we set out to address the lack of comprehensive search filters for immigrant populations in a health-related database by developing a robust search filter. However, the complexity of the topic lead us to create two filters to allow for a stronger capture of immigrant-related articles [68]. This approach provides searchers with the option to apply a desired level of specificity, precision, and sensitivity to their search. Searchers can select the specific filter, which maximizes specificity and precision, or increase sensitivity by adopting the sensitive filter. As with any comprehensive filter, our filters pulled many irrelevant studies or “noise” because of the inclusion of terms like “migration” which meets our inclusion criteria but also is used in non-immigrant related research.

Screening for the development set revealed that we could optimize sensitivity by adding language and cultural terms, which we opted to include as a sensitive filter. The need for supplemental filters further shows searching for studies on immigrant populations requires a multifaceted approach. Searchers must strategically build their search with filters such as the Immigrant Health search filters, the MEDLINE®/PubMed® Health Disparities and Minority Health Search Strategy while including other terms and concepts unique to their research questions [69]. In the future, we hope to see more systematic approaches to developing language and culture filters to enhance the sensitive filter to further optimize performance and improve search results.

As with any topic related to health, immigrant populations in health-related research are a nuanced subject leaving considerable room for subjectivity in the selection and relevance ranking of terms. We mitigated this as much as possible by creating clear eligibility criteria and a system for term identification and ranking. The exclusion of Rank 3 terms because they require multiple indicators means our filters missed relevant studies. Likewise, the filters did not find references with long phrases, such as “time living in the United States” that have countless iterations and are not recognized by PubMed's phrase index. Additional enhancements to PubMed, particularly the introduction of proximity searching, may make it more feasible to find records that use more complex word-phase combinations [43].

We also recognize a bias towards United States immigration in our selected terminology because of the heavy representation of U.S.-based researchers and publications in PubMed. To reduce the impact of geography bias, future development and review should incorporate collaborators from outside the U.S. to bring more global perspectives. Our commitment to the methodology and sourcing terms from pre-determined sources and building our reference and validation sets from topics based on local requests may have introduced selection bias and led to the omission of relevant word variants and terms. We hope to address this limitation in future versions of the filters by broadening the scope of authoritative sources and actively seeking input from researchers and fellow librarians. Likewise, publication of the filters paves the way for enhancements and refinements guided by input from peers, which will help rectify possible limitations stemming from the absence of peer review.

There is a need for collective consensus on reproducible search methodologies that can best help researchers to retrieve relevant literature about immigrants. Community search consortia in library professional groups, especially in countries that have national health systems, may also provide models for centralization and more international collaboration for the development, validation, and sharing of search filters, including those for immigrant populations. We await further developments in this area that will address not only the centralization aspects, but also the additional challenges of ensuring that search filter options can be discoverable not only by librarians but by researchers as well.

Developing the immigrant population filters was a three-year process. During that time, no other similarly focused search filters came to the forefront. The challenges we encountered, from trying to reduce bias and identify all relevant terms, to accommodating more nuanced concepts like language and culture, made us realize why comprehensive filters for immigrant populations have either not been developed or have not been widely shared. These factors motivated us to complete the project. Based on validation in two topic areas, we accomplished our goal to develop comprehensive search filters for immigrant populations to help find subsets of evidence in PubMed. Since the completion of the project, there have been notable advancements in Generative Artificial Intelligence and its associated tools with implications for search filter developments [70]. Further discussion on this subject exceeds the scope of this paper, as the report focuses on tools and approaches under consideration during the development and testing of the immigrant search filters. Nevertheless, we acknowledge the potential of generative AI for future iterations of the search filters. We look forward to future use, external testing, the possible expansion to other databases, and revisions using tools such as generative AI that would build upon our work and continue to improve the performance measurements thus making the specific and sensitive filters even more valuable to librarians and researchers.

## Data Availability

Associated data for this article are available at https://doi.org/10.18131/g3-163n-n075.

## SUPPLEMENTAL FILES

**Appendix A:** Immigrant Populations PubMed Search Filters
