# Supplementary material for: Meeting a need: development and validation of PubMed search filters for immigrant populations
Source: J Med Libr Assoc. 2024 Jan 16;112(1):22–32. doi: 10.5195/jmla.2024.1716 (PMC11189137; doi:10.5195/jmla.2024.1716)
Supplement: Supplementary file 1 — Appendix A: Immigrant Populations PubMed Search Filters [file jmla-112-1-22-s01.docx]

**APPENDIX A**

**Immigrant Populations PubMed Search Filters**

Developed by: Q. Eileen Wafford, MSt, MLIS, AHIP; Corinne Miller, MLIS; Annie Wescott, MLIS; Ramune K. Kubilius, MALS, AHIP

Affiliation: Northwestern University Fienberg School of Medicine Galter Health Sciences Library & Learning Center, Chicago, IL

Notes:

- Final, validated search filters for searching PubMed: Immigrant-specific filter and Immigrant-sensitive filter. Last updated November 2022.
- Future updated filters available at <https://doi.org/10.17605/OSF.IO/B6EMH>.

Citation: Wafford QE, Miller CH, Wescott AB, Kubilius RK. Immigrant Health PubMed Search Filters. Galter Health Sciences Library and Learning Center. 2022 Nov.

License**:** <http://creativecommons.org/licenses/by/4.0/>

**Immigrant-specific filter**

("Emigrants and Immigrants"[mesh] OR "Emigration and Immigration"[mesh] OR "Refugees"[mesh] OR "Transients and Migrants"[mesh] OR "Undocumented Immigrants"[mesh] OR "adopted country"[tiab] OR alien[tiab] OR aliens[tiab] OR asylee[tiab] OR asylees[tiab] OR asylum[tiab] OR "au pairs"[tiab] OR "au pair"[tiab] OR "birth abroad"[tiab] OR "birth countries"[tiab] OR "birth country"[tiab] OR "birth overseas"[tiab] OR "born abroad"[tiab] OR "born overseas"[tiab] OR "border communities"[tiab] OR "border community"[tiab] OR "border health"[tiab] OR "civil surgeon"[tiab] OR "civil surgeons"[tiab] OR citizenship[tiab] OR "countries of birth"[tiab] OR "country of birth"[tiab] OR "country of destination"[tiab] OR "countries of origin"[tiab] OR "country of origin"[tiab] OR "customs and border protection"[tiab] OR "deferred action for childhood arrivals"[tiab] OR daca[tiab] OR "department of homeland security"[tiab] OR deport[tiab] OR deportation[tiab] OR deportations[tiab] OR deported[tiab] OR "destination countries"[tiab] OR "destination country"[tiab] OR "diasporic communities"[tiab] OR "diasporic community"[tiab] OR "displaced person"[tiab] OR "displaced persons"[tiab] OR "displaced children"[tiab] OR "displaced families"[tiab] OR "displaced individuals"[tiab] OR "displaced men"[tiab] OR "displaced patients"[tiab] OR "displaced people"[tiab] OR "displaced peoples"[tiab] OR "displaced women"[tiab] OR "internationally displaced"[tiab] OR "duration of residence"[tiab] OR "ellis island"[tiab] OR emigrate[tiab] OR emigrated[tiab] OR emigration[tiab] OR emigrant[tiab] OR emigrants[tiab] OR emigrating[tiab] OR "exchange student"[tiab] OR "exchange students"[tiab] OR "exchange visitor"[tiab] OR "exchange visitors"[tiab] OR exile[tiab] OR exiled[tiab] OR exiles[tiab] OR expatriation[tiab] OR expatriate[tiab] OR expatriates[tiab] OR expatriated[tiab] OR "family reunification"[tiab] OR "family reunifications"[tiab] OR "first generation"[tiab] OR "first generations"[tiab] OR "2.5 generation"[tiab] OR "2.5 generations"[tiab] OR "second generations"[tiab] OR "third generation"[tiab] OR "third generations"[tiab] OR "mixed generation"[tiab] OR "foreign birth"[tiab] OR foreign-born[tiab] OR "foreign brides"[tiab] OR "foreign citizen"[tiab] OR "foreign citizens"[tiab] OR "foreign citizenship"[tiab] OR "foreign families"[tiab] OR "foreign family"[tiab] OR "foreign children"[tiab] OR "foreign men"[tiab] OR "foreign minor"[tiab] OR "foreign minors"[tiab] OR "foreign female"[tiab] OR "foreign females"[tiab] OR "foreign individuals"[tiab] OR "foreign male"[tiab] OR "foreign males"[tiab] OR "foreign patient"[tiab] OR "foreign patients"[tiab] OR "foreign people"[tiab] OR "foreign person"[tiab] OR "foreign persons"[tiab] OR "foreign resident"[tiab] OR "foreign residents"[tiab] OR "foreign national"[tiab] OR "foreign nationals"[tiab] OR "foreign nationalities"[tiab] OR "foreign nationality"[tiab] OR "foreign population"[tiab] OR "foreign populations"[tiab] OR "foreign woman"[tiab] OR "foreign women"[tiab] OR "foreigner"[tiab] OR "foreigners"[tiab] OR "green card"[tiab] OR "green cards"[tiab] OR "guest worker"[tiab] OR "guest workers"[tiab] OR "h 1b"[tiab] OR "host countries"[tiab] OR "host country"[tiab] OR "host societies"[tiab] OR "host society"[tiab] OR immigrant[tiab] OR immigrants[tiab] OR immigrate[tiab] OR immigrated[tiab] OR immigrates[tiab] OR immigrating[tiab] OR immigration[tiab] OR "intercountry adoption"[tiab] OR "intercountry adoptions"[tiab] OR "international adoption"[tiab] OR "international adoptions"[tiab] OR "international student"[tiab] OR "international students"[tiab] OR "length of residence"[tiab] OR "length of residency"[tiab] OR "maternal nativity"[tiab] OR migrant[tiab] OR migrants[tiab] OR migration[tiab] OR "migrated children"[tiab] OR nativity[tiab] OR naturalization[tiab] OR naturalized[tiab] OR "new countries"[tiab] OR "new country"[tiab] OR "new entrant"[tiab] OR "new entrants"[tiab] OR "new settler"[tiab] OR "new settlers"[tiab] OR "non citizen"[tiab] OR "non citizens"[tiab] OR noncitizen[tiab] OR noncitizens[tiab] OR "non national"[tiab] OR "non nationals"[tiab] OR nonnational[tiab] OR nonnationals[tiab] OR nonnative[tiab] OR nonnatives[tiab] OR "non native"[tiab] OR "non natives"[tiab] OR "non refoulement"[tiab] OR nonrefugee[tiab] OR nonrefugees[tiab] OR "overseas birth"[tiab] OR "overseas born"[tiab] OR "permanent residence"[tiab] OR "permanent residency"[tiab] OR "permanent resident"[tiab] OR "permanent residents"[tiab] OR "port health"[tiab] OR postmigration[tiab] OR premigration[tiab] OR "receiving countries"[tiab] OR "receiving country"[tiab] OR "receiving societies"[tiab] OR "receiving society"[tiab] OR refugee[tiab] OR refugees[tiab] OR "region of origin"[tiab] OR "regions of origin"[tiab] OR "resettlement countries"[tiab] OR "resettlement country"[tiab] OR "seasonal farmworkers"[tiab] OR "seasonal farm worker"[tiab] OR "seasonal farm workers"[tiab] OR "seasonal farmworker"[tiab] OR "seasonal worker"[tiab] OR "seasonal workers"[tiab] OR "stateless people"[tiab] OR "stateless person"[tiab] OR "stateless persons"[tiab] OR "stateless children"[tiab] OR "stateless individuals"[tiab] OR "stateless patients"[tiab] OR "temporary protected status"[tiab] OR "transnational community"[tiab] OR "unaccompanied child"[tiab] OR "unaccompanied children"[tiab] OR "unaccompanied minor"[tiab] OR "unaccompanied minors"[tiab] OR undocumented[tiab] OR UNHCR[tiab] OR visa[tiab] OR visas[tiab] OR "voluntary return"[tiab] OR xenophobia[tiab] OR xenophobic[tiab])

**Immigrant-sensitive filter**

("Emigrants and Immigrants"[mesh] OR "Emigration and Immigration"[mesh] OR "Refugees"[mesh] OR "Transients and Migrants"[mesh] OR "Undocumented Immigrants"[mesh] OR "adopted country"[tiab] OR alien[tiab] OR aliens[tiab] OR asylee[tiab] OR asylees[tiab] OR asylum[tiab] OR "au pairs"[tiab] OR "au pair"[tiab] OR "birth abroad"[tiab] OR "birth countries"[tiab] OR "birth country"[tiab] OR "birth overseas"[tiab] OR "born abroad"[tiab] OR "born overseas"[tiab] OR "border communities"[tiab] OR "border community"[tiab] OR "border health"[tiab] OR "civil surgeon"[tiab] OR "civil surgeons"[tiab] OR citizenship[tiab] OR "countries of birth"[tiab] OR "country of birth"[tiab] OR "country of destination"[tiab] OR "countries of origin"[tiab] OR "country of origin"[tiab] OR "customs and border protection"[tiab] OR "deferred action for childhood arrivals"[tiab] OR daca[tiab] OR "department of homeland security"[tiab] OR deport[tiab] OR deportation[tiab] OR deportations[tiab] OR deported[tiab] OR "destination countries"[tiab] OR "destination country"[tiab] OR "diasporic communities"[tiab] OR "diasporic community"[tiab] OR "displaced person"[tiab] OR "displaced persons"[tiab] OR "displaced children"[tiab] OR "displaced families"[tiab] OR "displaced individuals"[tiab] OR "displaced men"[tiab] OR "displaced patients"[tiab] OR "displaced people"[tiab] OR "displaced peoples"[tiab] OR "displaced women"[tiab] OR "internationally displaced"[tiab] OR "duration of residence"[tiab] OR "ellis island"[tiab] OR emigrate[tiab] OR emigrated[tiab] OR emigration[tiab] OR emigrant[tiab] OR emigrants[tiab] OR emigrating[tiab] OR "exchange student"[tiab] OR "exchange students"[tiab] OR "exchange visitor"[tiab] OR "exchange visitors"[tiab] OR exile[tiab] OR exiled[tiab] OR exiles[tiab] OR expatriation[tiab] OR expatriate[tiab] OR expatriates[tiab] OR expatriated[tiab] OR "family reunification"[tiab] OR "family reunifications"[tiab] OR "first generation"[tiab] OR "first generations"[tiab] OR "2.5 generation"[tiab] OR "2.5 generations"[tiab] OR "second generations"[tiab] OR "third generation"[tiab] OR "third generations"[tiab] OR "mixed generation"[tiab] OR "foreign birth"[tiab] OR foreign-born[tiab] OR "foreign brides"[tiab] OR "foreign citizen"[tiab] OR "foreign citizens"[tiab] OR "foreign citizenship"[tiab] OR "foreign families"[tiab] OR "foreign family"[tiab] OR "foreign children"[tiab] OR "foreign men"[tiab] OR "foreign minor"[tiab] OR "foreign minors"[tiab] OR "foreign female"[tiab] OR "foreign females"[tiab] OR "foreign individuals"[tiab] OR "foreign male"[tiab] OR "foreign males"[tiab] OR "foreign patient"[tiab] OR "foreign patients"[tiab] OR "foreign people"[tiab] OR "foreign person"[tiab] OR "foreign persons"[tiab] OR "foreign resident"[tiab] OR "foreign residents"[tiab] OR "foreign national"[tiab] OR "foreign nationals"[tiab] OR "foreign nationalities"[tiab] OR "foreign nationality"[tiab] OR "foreign population"[tiab] OR "foreign populations"[tiab] OR "foreign woman"[tiab] OR "foreign women"[tiab] OR "foreigner"[tiab] OR "foreigners"[tiab] OR "green card"[tiab] OR "green cards"[tiab] OR "guest worker"[tiab] OR "guest workers"[tiab] OR "h 1b"[tiab] OR "host countries"[tiab] OR "host country"[tiab] OR "host societies"[tiab] OR "host society"[tiab] OR immigrant[tiab] OR immigrants[tiab] OR immigrate[tiab] OR immigrated[tiab] OR immigrates[tiab] OR immigrating[tiab] OR immigration[tiab] OR "intercountry adoption"[tiab] OR "intercountry adoptions"[tiab] OR "international adoption"[tiab] OR "international adoptions"[tiab] OR "international student"[tiab] OR "international students"[tiab] OR "length of residence"[tiab] OR "length of residency"[tiab] OR "maternal nativity"[tiab] OR migrant[tiab] OR migrants[tiab] OR migration[tiab] OR "migrated children"[tiab] OR nativity[tiab] OR naturalization[tiab] OR naturalized[tiab] OR "new countries"[tiab] OR "new country"[tiab] OR "new entrant"[tiab] OR "new entrants"[tiab] OR "new settler"[tiab] OR "new settlers"[tiab] OR "non citizen"[tiab] OR "non citizens"[tiab] OR noncitizen[tiab] OR noncitizens[tiab] OR "non national"[tiab] OR "non nationals"[tiab] OR nonnational[tiab] OR nonnationals[tiab] OR nonnative[tiab] OR nonnatives[tiab] OR "non native"[tiab] OR "non natives"[tiab] OR "non refoulement"[tiab] OR nonrefugee[tiab] OR nonrefugees[tiab] OR "overseas birth"[tiab] OR "overseas born"[tiab] OR "permanent residence"[tiab] OR "permanent residency"[tiab] OR "permanent resident"[tiab] OR "permanent residents"[tiab] OR "port health"[tiab] OR postmigration[tiab] OR premigration[tiab] OR "receiving countries"[tiab] OR "receiving country"[tiab] OR "receiving societies"[tiab] OR "receiving society"[tiab] OR refugee[tiab] OR refugees[tiab] OR "region of origin"[tiab] OR "regions of origin"[tiab] OR "resettlement countries"[tiab] OR "resettlement country"[tiab] OR "seasonal farmworkers"[tiab] OR "seasonal farm worker"[tiab] OR "seasonal farm workers"[tiab] OR "seasonal farmworker"[tiab] OR "seasonal worker"[tiab] OR "seasonal workers"[tiab] OR "stateless people"[tiab] OR "stateless person"[tiab] OR "stateless persons"[tiab] OR "stateless children"[tiab] OR "stateless individuals"[tiab] OR "stateless patients"[tiab] OR "temporary protected status"[tiab] OR "transnational community"[tiab] OR "unaccompanied child"[tiab] OR "unaccompanied children"[tiab] OR "unaccompanied minor"[tiab] OR "unaccompanied minors"[tiab] OR undocumented[tiab] OR UNHCR[tiab] OR visa[tiab] OR visas[tiab] OR "voluntary return"[tiab] OR xenophobia[tiab] OR xenophobic[tiab])

OR

("Culture"[Mesh] OR culture[tiab] OR culturally[tiab] OR cultures[tiab] OR acculturated[tiab] OR acculturates[tiab] OR acculturation[tiab] OR acculturations[tiab] OR assimilation[tiab] OR assimilated[tiab] OR bicultural[tiab] OR cultural[tiab] OR ethniccultural[tiab] OR ethnocultural[tiab] OR intercultural[tiab] OR multicultural[tiab] OR multiculturalism[tiab] OR sociocultural[tiab] OR socioculturally[tiab] OR transcultural[tiab])

OR

(Language[ti] OR "language assistance"[tiab] OR "language isolation"[tiab] OR "primary language"[tiab] OR "language spoken"[tiab] OR "second language"[tiab] OR "native language"[tiab] OR "native languages”[tiab] OR "minority languages”[tiab] OR "minority language"[tiab] OR (language[tiab] AND speaking[tiab]) OR "language related differences"[tiab] OR "language proficiency"[tiab] OR "english proficiency"[tiab] OR "language fluency"[tiab] OR "patient language"[tiab] OR "language access services"[tiab] OR "language needs"[tiab] OR linguistic*[tiab] OR "limited English proficiency"[tiab] OR multilingual*[tiab] OR "multi language"[tiab] OR "communication gap"[tiab] OR "medical interpretation"[tiab] OR interpreter*[tiab] OR "medical translation"[tiab] OR "medical translator"[tiab] OR "medical translators"[tiab] OR "limited English proficiency"[tiab] OR "limited English proficient"[tiab] OR LEP[tiab] OR "non English speaking"[tiab] OR "non English speaker"[tiab] OR "non English speakers"[tiab] OR "translated materials"[tiab] OR NESB[tiab] OR "remote simultaneous interpretation"[tiab] OR ((interpretation[tiab] OR translation[tiab] OR interpreter[tiab] OR interpreters[tiab] OR translator[tiab] OR translators[tiab] OR barrier[tiab] OR barriers[tiab] OR discordance[tiab] OR discordances[tiab] OR concordance[tiab] OR concordances[tiab]) AND (language[tiab] OR consultation*[tiab] OR communication[tiab])) )
